# A novel method for isolation and flow cytometry analysis of intraepithelial lymphocytes from colon biopsies

**DOI:** 10.3168/jdsc.2022-0352

**Published:** 2023-04-28

**Authors:** K. Lamers, M.A. Steele, L.R. Cangiano

**Affiliations:** Department of Animal Biosciences, University of Guelph, Guelph, ON, N1G 2W1, Canada

## Abstract

Investigating the immune responses of the intestine in response to different insults is predominantly limited to indirect methods such as circulating markers of intestinal health or gene expression from dissections. We describe here a validated protocol for the isolation and subsequent flow cytometry analysis of intestinal intraepithelial lymphocytes (IEL) from colonic biopsy samples. Colon biopsy samples were collected with endoscopy forceps from Holstein dairy bull calves at d 2, 28, and 42 of life. The biopsies were put into an isolation solution of Hanks' balanced salt solution, and fetal bovine serum followed by digestion solution. The solution was filtered and the flow-through, containing IEL, was stained with fluorescent antibodies for flow cytometry analysis. Density gradient separation of the isolate yielded higher viability and cleaner samples for flow cytometry analysis. Anti-bovine γ chain of the T cell receptor was used to identify populations of gamma delta (γδ) T cells via flow cytometry. In addition, γδ T cell subsets were identified using an anti-bovine antibody against the coreceptor workshop cluster 1. This method allowed for the precise identification of lymphocyte populations and evaluation of the proportion of different subsets of γδ T cells from intestinal IEL over time. The technique described here will allow the research community to characterize intestinal immune function over time and improve our understanding of how different management and nutritional strategies affect intestinal health.

## Highlights

• Colonic epithelial lymphocytes can be isolated from biopsy samples.
• Using a density gradient separation step during intestinal epithelial lymphocyte isolation improves the sample quality.
• Gamma delta T cells and WC1.2+ T cell colon populations increase with age in Holstein calves.

## Graphical Abstract

**Figure:**
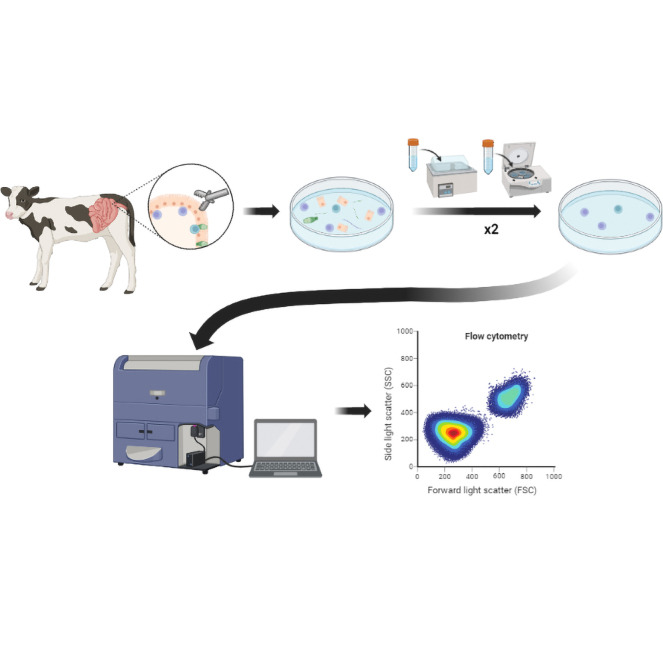
**Summary:** This technical note details a methodology for the isolation of intestinal epithelial lymphocytes from colon biopsy samples in calves. Intestinal epithelial lymphocytes were isolated through a series of incubation and centrifugation steps, followed by density gradient separation. The lymphocytes were then stained with gamma delta T cell antibodies to measure the proportions of these cells over time via flow cytometry. This will improve our ability to study intestinal immune function during health and disease.

The intestinal epithelia of the small and large intestine serve the dual purpose of absorbing nutrients while preventing intestinal microorganisms from breaching this anatomical barrier (Cangiano et al., 2022). Several intestinal disorders and pathological conditions stem from a dysfunctional gastrointestinal barrier, leading to increased morbidity (Mani et al., 2012). This is particularly important in calves, as gastrointestinal disorders account for over one-half of the reported illnesses and one-third of deaths, and are the leading cause of morbidity and mortality during the preweaning period (Urie et al., 2018). Furthermore, metabolic and digestive disorders are some of the most common reasons why dairy cattle are culled from the lactating herd, and impaired barrier function has been associated with various metabolic diseases in dairy cattle (Rilanto et al., 2020; Sanz-Fernandez et al., 2020). The intestinal barrier is made up of layers of mucus that prevent direct exposure of the epithelial cells to the intestinal milieu. This intestinal epithelial cells form a tight monolayer that prevents infiltration of unwanted compounds (Vancamelbeke and Vermeire, 2017). Numerous different cell types form the intestinal epithelium including absorptive enterocytes, goblet cells, Paneth cells, microfold cells, and intraepithelial lymphocytes (**IEL**; Farré et al., 2020). Beneath the epithelium lies the lamina propria, a loose connective tissue containing intestinal lymphocytes and other immune mediators, such as complement, chemokines, and cytokines (Mowat and Agace, 2014; Chase, 2018).

The gastrointestinal immune system is the largest immune compartment in the body and with spatial and regional differences in function to meet the needs of each compartment of the small and large intestine (Mowat and Agace, 2014; Agace and McCoy, 2017). The immune system of the gastrointestinal tract must tightly regulate tolerance to commensal bacteria and harmless antigens while maintaining the ability to respond against microbial infections (Ma et al., 2021). Dysregulation of these critical functions can result in increased susceptibility to intestinal pathogens and promote aberrant immune responses that lead to intestinal inflammation and tissue damage (Chase, 2018). Tolerance to food and commensal microbiota-derived antigens is critical to maintaining homeostasis in the gastrointestinal tract. In calves, a population of T cells, called gamma delta T cells (γδ T cells), make up to 60% of circulating lymphocytes at birth. Gamma delta T cells recognize antigens with the workshop cluster (**WC^+^**) coreceptor that is further divided into 2 subsets: WC1.1^+^ and WC1.2^+^. The WC1.1^+^ subset is involved in clearing viral infections and, upon activation, secretes proinflammatory molecules as well as granzymes and perforin (Baldwin et al., 2021). Circulating γδ T cells that constitutively express the coreceptor WC1.2^+^ and WC1^−^ cells, primarily found in the intestine, have been shown to have an immunomodulatory role both in vivo and in vitro (Guzman et al., 2014). Gamma delta T cells play a critical role in maintaining immune homeostasis and preventing infection early in life, particularly at a time when maternal passive immunity starts to decline, but the adaptive immune system of the calf is not properly equipped to mount adequate responses to clear infections (Chase et al., 2008). Few studies have measured resident intestinal lymphocyte populations over time or studied the developmental changes that occur in calves. Tissue sampling of the bovine small and large intestine have been limited so far to cannulation or slaughter. Investigating the immune response to gut pathogens is predominantly limited to indirect methods such as circulating cytokine expression or tissue gene expression. The objective of this study was to develop a method of isolating lymphocytes from the intestinal epithelium of biopsies collected from dairy calves and to evaluate the proportions of colonic lymphocyte subsets over time.

A total of 18 Holstein bull calves were obtained from the Elora Dairy Research Station and housed in individual pens at the Ponsonby general animal facility at the University of Guelph (Guelph, Canada). The experiment was conducted per the guidelines of the Canadian Council of Animal Care at the Ponsonby Research Station, University of Guelph (Guelph, ON, Canada). The animal use protocol was approved by Animal Care Committee at the University of Guelph (Animal Use Protocol #4470).

Colon tissue biopsies were taken according to the procedure described by van Neikerk et al. (2018). Briefly, calves were restrained in a calf chute, and lubricant was applied to an endoscope before insertion. The endoscope (160 cm length, 9.8 mm diameter; GIF-Q140, Olympus) connected to a light source and processor (CLV-U40 and CV-140, Olympus) was slowly inserted through the calf's rectum until 60 to 80 cm past the anal orifice. Endoscopic biopsy forceps (MultiCROC biopsy forceps, 2.4 mm diameter; Primed Instruments) were used to collect 20 colon tissue samples. The samples were placed in PBS, then transferred to 4°C Hanks' balanced salt solution (**HBSS**; calcium, magnesium, and phenol red-free; SH30588.02, Cytiva) for transportation back to the laboratory (Figure 1A). The biopsy samples were then transferred into 10 mL of isolation solution, made up of HBSS supplemented with 10% HEPES (B299–1, Fisher), 4% heat-inactivated fetal bovine serum (26010066, Fisher), and 5 m*M* EDTA (V4233; Promega). Samples were then vortexed before incubation for 20 min at 37°C, with additional vortexing every 5 min during incubation. After incubation, the tubes were vortexed thoroughly and the solution was filtered through a 100-μm cell strainer (352360, Corning). The flow through, containing IEL, was kept. The process was repeated twice, and the remaining biopsy tissue was placed in 5 mL of digestion solution, made up of Roswell Park Memorial Institute (RPMI) 1640 media (11875093, Fisher) supplemented with 10% fetal bovine serum, 1 mg/mL collagenase (type VIII; C2139, Sigma), 10 U/mL DNase, and 10 m*M* HEPES. The biopsies were vortexed, then incubated at 37°C for another 20 min, and 5 mL of HBSS was added to the tube at the end of the incubation to stop enzymatic digestion. The solution was filtered through a 100-μm cell strainer and the flow-through was centrifuged at 400 × *g* for 5 min at room temperature (**RT**). The supernatant was discarded, and cells were washed with 5 mL of HBSS, centrifuged at 400 × *g* for 5 min at RT (22°C), and resuspended in 500 µL of PBS. Cells were then counted using Trypan blue (15250061, Fisher). The IEL were found to be very sticky, with doublets or groups of cells attached to each other; however, this did not appear to affect their viability (Table 1). After isolation, we proceeded to stain cells for flow cytometry analysis.

**Figure 1:**
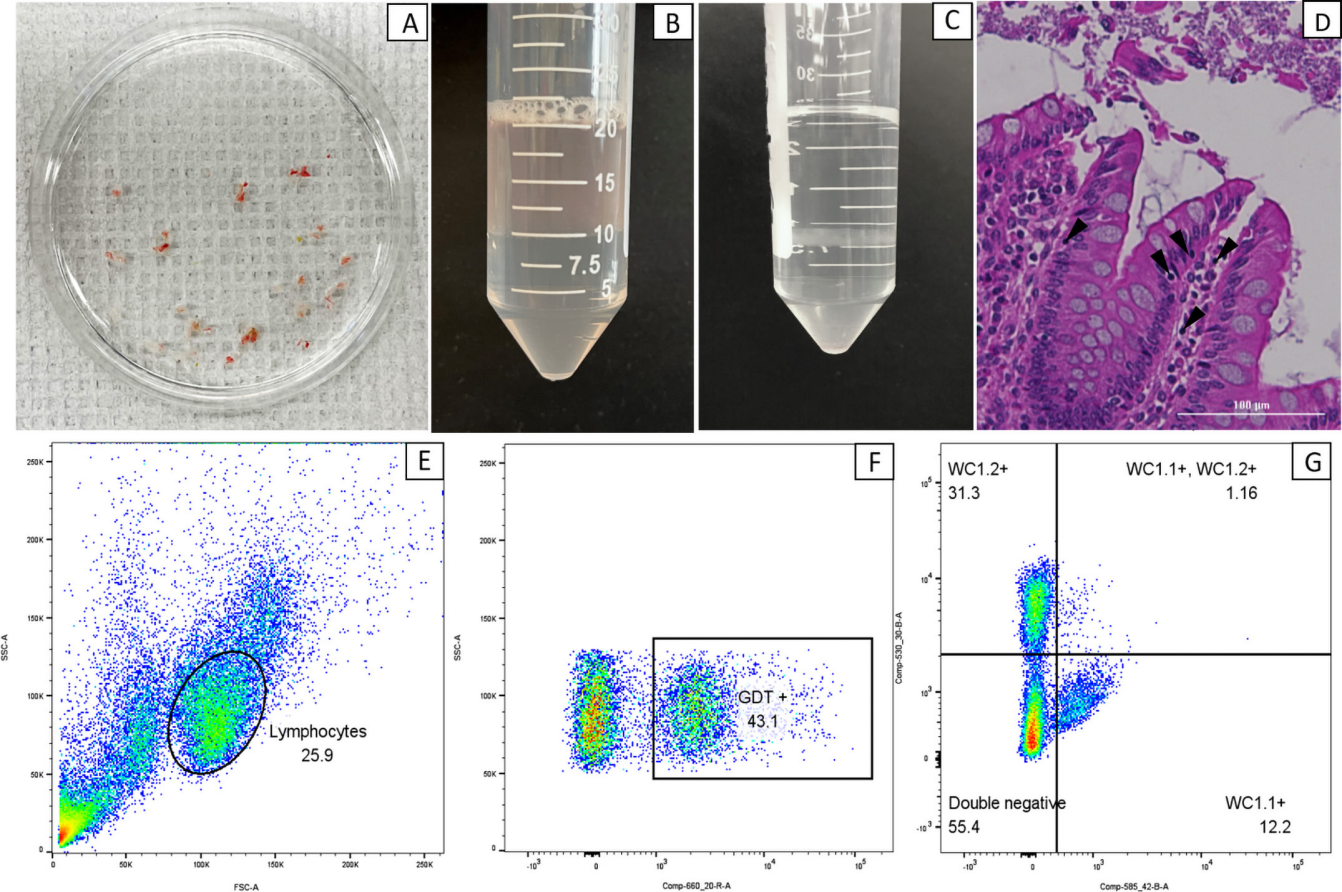
Isolation and flow cytometry of colon biopsies from Holstein calves. (A) Freshly sampled biopsies in Hanks' balanced salt solution. (B) Postisolation solution cell suspension layered on top of Histopaque (Sigma). (C) Density gradient media separated cell suspension; note the interphase layer. (D) Hematoxylin and eosin stain of colon tissue collected from a 42-d-old calf. 20× Brightfield image. Arrows indicate leukocytes within the epithelium. (E) Intraepithelial lymphocyte (IEL) gating. (F) Gamma delta (γδ) T cell (GDT) gating of IEL. (G) WC1.1 and WC1.2 gating of γδ IEL. FSC = forward light scatter; SSC = side light scatter.

**Table 1:** Cell viability of colonic biopsy intraepithelial lymphocytes using varying isolation techniques (means ± SEM)

Isolation method	% of cell viability
Mechanical/chemical digestion only (digestion)	75.6 ± 8.4
Digestion + density gradient separation	78.2 ± 9.0
Digestion + density gradient separation + TrypLE Express^1^	67.8 ± 16.7

1 Thermo Fisher Scientific.

The cells were stained with 1:400 (stock concentration 1 µg/μL) of a GB21A anti-bovine γδ T cell antibody against the γ chain of the γδ T cell receptor (Washington State University) for 30 min in the dark at RT. During incubation, the secondary antibodies were prepared in separate tubes. The secondary solution contained 100 μL of 1:400 dilution anti-mouse IgG2b conjugated to APC (1090–11S; Southern Biotech). The next solution contained 1 μL of anti-WC1.1 antibody (CACTB32A; Washington State University) in 25 μL of PBS with 4 μL of anti-mouse IgG1 zenon RPE labeling solution and after 5 min of incubation 4 μL of quench from the same kit (Z25055, Fisher) was added. The final secondary solution contained 1 μL of anti-WC1.2 antibody (BAQ159A, Washington State University) with 4 μL of anti-mouse IgG1 zenon AlexaFluor 488 labeling solution and after 5 min of incubation 4 μL of quencher solution (Z25002, Fisher) was added. After incubation, 300 μL of PBS with 0.5% BSA (**PBA**) was added, then the solution was centrifuged at 700 × *g* for 5 min at RT. The supernatant was discarded, and the cells were washed twice with PBA. The pellet was then resuspended in a solution of the secondary antibodies, and incubated for 30 min in the dark at RT, then 300 μL of PBA was added and washed twice with PBA.

The stained cells were resuspended in 200 μL of PBS before running on a BD FACSCanto (BD Biosciences). A total of 10,000 events were collected per sample. The first few samples had contamination with nonleukocytic cell populations and tissue debris with a low proportion of IEL compared with total events. We opted to add a density gradient separation step after the initial isolation steps with isolation solution and digestion solution to allow for a better separation between lymphocytes and the rest of cells present in the media. The cell suspension solutions were centrifuged at 400 × *g* for 5 min at RT. The supernatant was discarded, then the cells were resuspended in 10 mL of PBS. Histopaque-1077 (10771; Sigma) was slowly added to the bottom of the tube using a 20-mL syringe and a 18-gauge spinal needle (Spinal NRFit, BD Biosciences; see Figure 1B) and the tube was then centrifuged at 1,200 × *g* for 15 min at RT. The interphase layer containing lymphocytes was collected from the tubes (Figure 1C), topped up with PBS to 30 mL, then centrifuged at 400 × *g* for 5 min at RT. The supernatant was discarded, and the cells were then resuspended in 500 µL of PBS and counted using Trypan blue and stained for flow cytometry as previously described. The density gradient separation step improved the proportion of lymphocytes in the sample, though this difference was not statistically significant (Table 1). Doublets were found during the cell counting step. To overcome this issue, we tested an extra incubation step with TrypLE Express (12605010, Thermo Fisher Scientific) at 37°C for 5 min, immediately followed by the addition of 15 mL of PBS. We did not observe a significant reduction in presence of aggregated and doublet cells in the IEL samples; however, it increased cell lysing and decreased viability, though this was not found to be statistically significant (Table 1).

Flow cytometry data were analyzed with FlowJo (FlowJo LLC), using unstained samples and fluorescence minus one samples to establish appropriate gating. Additionally, compensation beads (UltraComp eBeads Plus microspheres, Thermo Fisher) were used to calculate spillover values and set appropriate voltages and gating parameters. Data were analyzed using a generalized linear mixed model to assess the effect of isolation method and effect of day. Data were assessed for normality with the Jarque-Bera normality test. The *P*-values were adjusted with Tukey for multiple comparisons. All cell markers used in this protocol were successfully detected by flow cytometry with good sensitivity. We observed an increase in the proportion of γδ T cells (*P* = 0.03; Table 2) within the IEL and a tendency for WC1.2^+^ γδ T cells to increase over time with greater proportions of WC1.2^+^ γδ T cells on d 42 compared with d 2 of life (*P* = 0.05; Table 2). No differences were observed for WC1.1^+^ γδ T cells.

**Table 2:** Colonic biopsy isolated intraepithelial lymphocyte population composition by age in Holstein dairy bull calves [means and standard error of the difference (SED)]

Population, %	Days			SED	*P*-value
	2	28	42		Day effect
γδ T cell lymphocytes^1^	30.5^a^	38.2^ab^	43.6^b^	4.58	0.03
WC1.1^+^ γδ T cells^2^	13.7	4.7	12.7	6.71	0.38
WC1.2^+^ γδ T cells^2^	4.7ª	11.6^ab^	14.8^b^	4.08	0.06

a,b Different superscripts denote significant differences (*P* < 0.05) between days assessed by generalized linear mixed model.

1 Proportion of gamma delta T cells (γδ T cells) within the lymphocyte population based on the expression of the γδ T cell receptor (γδTCR^+^ lymphocytes).

2 Proportion of γδTCR^+^ lymphocytes that express the workshop cluster (WC) coreceptors: WC1.1^+^ or WC1.2^+^.

The increase in the proportion of γδ T cells in the colon is consistent with a study that isolated IEL from ileal intestinal segments from postmortem calves, which observed a gradual increase in γδ T cells from 1-wk-old calves (28 ± 2%) to 6- to 9-wk-old calves (50 ± 7%; Parsons et al., 1996). This trend in increasing IEL γδ T cells in calves of increasing age is also consistent with another study that observed a similar trend, though at lower proportions (Wyatt et al., 1996). Last, although the isolation method used should yield primarily IEL, we cannot discard the possibility that some lamina propria lymphocytes might have been present in the sample.

Under normal conditions, the intestinal epithelium and mucosal layers act as gatekeepers, regulating the entrance of nutrients while preventing the access of microbes into circulation (Steele et al., 2016). Furthermore, the intestinal immune compartment is the largest in the body and mediates tolerance toward commensal bacteria and dietary antigens while preserving the ability to mount immune responses in the face of microbial infections (Mani et al., 2012). In response to different challenges, the intestinal barrier function can be compromised leading to aberrant immune responses, tissue damage, and systemic inflammation (Vanuytsel et al., 2021; Cangiano et al., 2022). To date, studies of intestinal immune function have been limited to indirect measurements of intestinal health, or direct tissue sampling via cannulation or euthanasia. The technique described here can provide a tool to better understand how our current management practices affect colon mucosal immunity in cattle during health and diseased states. Furthermore, it will enable the development of longitudinal studies to understand what environmental factors can affect the development of the intestinal immune compartment in early life. However, data should be interpreted with caution, as immune cell populations observed using this technique might not be representative of the overall immune status of the intestine, and therefore interpretation should be constrained to the intestinal location from where the samples were collected. This is a relatively noninvasive procedure that can grant repeated sampling over time, as well as prevent the euthanasia of healthy animals, which in turn may allow for the use of smaller sample sizes. The use of longer endoscopes and improvements in the sampling technique could be used in the future to sample from the proximal colon and ileum. Last, flow cytometry analysis has the benefit of identifying multiple cell populations in real time, which can be used to infer function based on cell surface or intracellular markers.

The technique described here has very clear advantages over other direct and indirect methods to study intestinal immunity and gastrointestinal health with multiple applications in our field. However, there are a few technical challenges to be considered. To get a significant number of IEL, a relatively large number of biopsies must be taken, which can be challenging with neonatal calves, and might induce inflammation at the site of sampling. It is possible that fewer samples would be required if sampling from the ileum, due to the increased abundance of lymphocytes in this region of the intestine compared with the colon (Chase and Kaushik, 2019). Though our technique allows for multiple sampling times, these periods should be spaced at least 2 wk apart from each other to allow for healing, based on recommendations from human clinical trials (Subramanyam et al., 1985). Last, although we did not observe changes in cell surface expression of the cell surface markers measured in this study, enzymatic digestion has been previously reported to affect cell surface marker expression (Trapecar et al., 2017). Further research will be required to determine if IEL extracted from the colon are representative of the overall immune status of the intestine.

In summary, IEL can be successfully isolated from endoscopic colon biopsies with sufficient viability for flow cytometry analysis. The addition of a density gradient separation step improved sample viability and cleanliness by removing many nonviable cells and debris. Isolation and staining of IEL can be used to further assess and evaluate intestinal health in cattle. Assessments that can directly appraise the function of the gastrointestinal tract immune system provide an important tool to better understand how current management conditions and nutritional strategies affect the intestinal health of dairy cattle.
